# Draft Genome Sequences of *Paenibacillus polymyxa* NRRL B-30509 and *Paenibacillus terrae* NRRL B-30644, Strains from a Poultry Environment That Produce Tridecaptin A and Paenicidins

**DOI:** 10.1128/genomeA.00372-15

**Published:** 2015-04-23

**Authors:** Marco J. van Belkum, Christopher T. Lohans, John C. Vederas

**Affiliations:** Department of Chemistry, University of Alberta, Edmonton, Alberta, Canada

## Abstract

*Paenibacillus polymyxa* NRRL B-30509 and *Paenibacillus terrae* NRRL B-30644 produce tridecaptin A that is inhibitory to *Campylobacter jejuni*, as well as lantibiotics in the paenicidin family. Here, we report the draft genome sequences of *P. polymyxa* NRRL B-30509 and *P. terrae* NRRL B-30644 that contain gene clusters for various nonribosomal lipopeptides.

## GENOME ANNOUNCEMENT

*Campylobacter jejuni* is a leading cause of gastroenteritis (1). Infection with *Campylobacter* occurs primarily through the consumption and handling of poultry. Antibiotic resistance of *Campylobacter* strains is a concern, and recently, attention has focused on the isolation of peptides that are active against *Campylobacter* species*. Paenibacillus polymyxa* NRRL B-30509 and *Bacillus circulans* NRRL B-30644, isolated from a poultry production environment, were reported to produce bacteriocins inhibitory to *C. jejuni* (2). However, subsequent studies showed no evidence that these bacteriocins were produced, and instead the activity in both strains against *C. jejuni* was attributed to the nonribosomal lipopeptide tridecaptin A (3, 4). In addition, NRRL B-30509 and NRRL B-30644 were found to produce the novel lantibiotics paenicidin A and B, respectively (3, 4). Based on 16S rRNA analysis, *B. circulans* NRRL B-30644 was renamed *Paenibacillus terrae* NRRL B-30644 (4).

Genomic DNA of *P. polymyxa* NRRL B-30509 and *P. terrae* NRRL B-30644 were isolated using the DNeasy blood & tissue kit (Qiagen) and sequenced by 454 GS FLX Titanium pyrosequencing (Roche) at GenoSeq (UCLA Genotyping and Sequencing Core, Los Angeles, CA). The reads were assembled into contigs using the GS De Novo Assembler software (Roche). The assembly of the draft genome sequence of *P. polymyxa* NRRL B-30509 yielded 75 contigs (>200 bp) and contains 5,948,280 bases with a G+C content of 45.1%. For *P. terrae* NRRL B-30644, the assembled draft genome sequence consists of 234 contigs (>200 bp) and 6,514,862 bases with a G+C content of 45.7%. One of these 234 contigs carries the genes for tridecaptin A production and was assembled from different contigs previously (4). Genome annotation by the NCBI Prokaryotic Genomes Automatic Annotation Pipeline (PGAAP) predicted 5,169 genes, including 4,839 coding sequences (CDS), 33 rRNAs, and 109 tRNAs in the genome of NRRL B-30509, and 5,988 genes, including 5,482 CDS, 14 rRNAs, and 95 tRNAs in the genome of NRRL B-30644.

The average nucleotide identity (ANI) between *P. polymyxa* NRRL B-30509, *P. terrae* NRRL B-30644, *P. polymyxa* SC2 (5), and *P. terrae* HPL-003 (6) was calculated using JSpecies software (7). NRRL B-30509 has a higher identity to SC2 (94.71% ANI) than to NRRL B-30644 and HPL-003 (84.82% and 84.68% ANI, respectively), whereas NRRL B-30644 has a higher identity to HPL-003 (93.28% ANI) than to NRRL B-30509 and SC2 (84.75% and 84.54% ANI, respectively). This is in agreement with the previous finding that NRRL B-30644 is a *P. terrae* species (4).

*P. polymyxa* NRRL B-30509 carries gene clusters for several nonribosomal lipopeptides, including tridecaptin A, polymyxin, and fusaricidin. Indeed, it was previously observed that NRRL B-30509 also produces polymyxins E1 and E2 (3). The fusaricidin synthetase gene in NRRL B-30509 contains a frameshift mutation, which might prevent the production of fusaricidin. In addition to the paenicidin A gene cluster, NRRL B-30509 also harbors a lantibiotic gene cluster that might produce a novel subtilin-like bacteriocin. The genome of *P. terrae* NRRL B-30644 carries the gene clusters for tridecaptin A and fusaricidin, as well as for paenicidin B.

### Nucleotide sequence accession numbers. 

These whole-genome shotgun projects have been deposited at DDBJ/EMBL/GenBank under the accession numbers JTHO00000000 for *P. polymyxa* NRRL B-30509 and JTHP00000000 for *P. terrae* NRRL B-30644. The versions described in this paper are the first versions, JTHO01000000 and JTHP01000000, respectively.
